# Sexual aggressors with mental disorders: characterization of a Tunisian sample

**DOI:** 10.1192/j.eurpsy.2023.1861

**Published:** 2023-07-19

**Authors:** A. Chamseddine, N. Charfi, S. Ajmi, I. Gassara, N. Smaoui, R. Feki, S. Omri, L. Zouari, J. Ben Thabet, M. Maâlej Bouali, M. Maâlej

**Affiliations:** Psychiatry C, Hedi Chaker Hospital, Sfax, Tunisia

## Abstract

**Introduction:**

Sexual violence is a worldwide public health concern. Nevertheless, the psychopathology of perpetrators of sexual assault still nowadays poorly documented.

**Objectives:**

The aim of this study was to assess the characteristics of sexual aggressors with mental disorders.

**Methods:**

It was a retrospective study that included a series of sexual aggressors examined in forensic psychiatric assessment in the psychiatry C department at Hedi Chaker university Hospital in Sfax, from January 2010 to December 2021. Data were collected from psychiatric expert reports.

**Results:**

The sample was exclusively composed of men with an average age of 37 years and 07 months (± 12.75 years). 54.4% of sexual aggressors suffered from mental disorder. Personality disorder was the most prevalent psychiatric disorder and the antisocial type was noted in 23.9% of cases.

Sexual aggressors suffering from mental disorder were more likely to commit rape followed by murder (p=0.05). They used physical violence far more than the others did (p=0.007) and they were more apt to threaten their victims with weapon during the assault (p=0.038). They were also more likely to abuse the power given by their professional roles (15,2 % versus 5,6 %; p=0.07). They more frequently attacked unknown victims (p=0.019).

**Conclusions:**

More than half of sexual aggressors suffered from psychiatric disorder. Therefore, the detection and treatment of psychiatric morbidity among sexual aggressors may minimize the risk of recidivism.

**Disclosure of Interest:**

None Declared

